# The Effects of 12-Weeks Whey Protein Supplements on Markers of Bone Turnover in Adults With Abdominal Obesity – A *Post Hoc* Analysis

**DOI:** 10.3389/fendo.2022.832897

**Published:** 2022-03-29

**Authors:** Rasmus Fuglsang-Nielsen, Elin Rakvaag, Peter Vestergaard, Kjeld Hermansen, Søren Gregersen, Jakob Starup-Linde

**Affiliations:** ^1^Department of Endocrinology and Internal Medicine, Aarhus University Hospital, Aarhus, Denmark; ^2^Steno Diabetes Center Aarhus, Aarhus University Hospital, Aarhus, Denmark; ^3^Steno Diabetes Center North Jutland, Aalborg University Hospital, Aalborg, Denmark; ^4^Department of Endocrinology, Aalborg University Hospital, Aalborg, Denmark; ^5^Department of Clinical Medicine, Aalborg University, Aalborg, Denmark; ^6^Department of Clinical Medicine, Aarhus University, Aarhus, Denmark

**Keywords:** bone turnover, abdominal obesity, dietary protein, dietary fiber, insulin resistance

## Abstract

**Background:**

While osteoporosis is characterized by skeletal fragility due to increased bone turnover and low bone mineral density (BMD), subjects with abdominal obesity and type-2 diabetes have increased risk of bone fractures despite low bone turnover and increased BMD. Diets with increased protein content are reported to increase bone turnover in healthy adults and may be a point of interest in preserving bone strength in subjects with abdominal obesity and/or type-2 diabetes.

**Methods:**

We examined the effect of 12-weeks dietary intervention on bone turnover in 64 adults with abdominal obesity using data from the MERITS trial. The trial was a randomized, controlled, double blinded study in which participants were allocated to receive either 60 g/d of whey protein hydrolysate or maltodextrin in combination with either high (30 g/d) or low dietary fiber intake (10 g/d). Primarily, we assessed changes in plasma markers of bone turnover Procollagen type 1 N-terminal propeptide (p1NP), C-terminal telopeptide type-1 collagen (CTX), and parathyroid hormone (PTH) within the four intervention groups. In addition, we measured u-calcium and u-carbamide excretion, 25(OH)D, and BMD by whole body DXA scans. Finally, we compared changes in insulin resistance (Homeostasis-model assessment of insulin resistance, HOMA-IR) with changes in bone turnover markers.

The trial was registered at www.clinicaltrials.gov as NCT02931630.

**Results:**

Sixty-four subjects were included in the study. We did not find any effect of twelve weeks of high protein or high fiber intake on plasma levels of P1NP or CTX. There was a nonsignificant positive association between protein intake and PTH levels (p=0.06). U-calcium and u-carbamide increased in both protein groups.

There was a positive association between change in HOMA-IR and PTH (p=0.042), while changes in P1NP and CTX did not associate to changes in HOMA-IR.

**Conclusion:**

Twelve weeks of increased whey protein intake in subjects with abdominal obesity did not affect markers of bone turnover significantly, although tended to increase PTH levels. Dietary fiber intake did not affect bone turnover. We report a positive association between change in HOMA-IR and PTH supporting a hypothesis of insulin resistance as a potential key factor in the expanding field of bone fragility in T2D subjects.

## Introduction

A diet rich in protein may be beneficial to skeletal properties, since bone structure largely consists of proteins integrated within the organic collagen matrix during mineralization. Nevertheless, the effect of high protein diets remains poorly established when it comes to bone health (1).

Most studies on protein diets and bone health are performed on healthy or postmenopausal women, where an increased risk of fractures relates to increased bone turnover with resulting loss of bone mass. However, in dysmetabolic conditions dominated by insulin resistance such as abdominal obesity, metabolic syndrome or prediabetes and type 2 diabetes, the determination of bone properties seems to be quite different. Contrasting to postmenopausal osteoporosis, bone health is dominated by low bone turnover (2–7), which may lead to unrepaired micro-fractures and poor bone quality (8).

Formerly, obesity was considered a protective feature against fractures due to mechanical protective properties of subcutaneous fat in the hip region and an increased bone density from increased mechanical load. Within the last decades, this assumption has been challenged (9, 10). Although body mass index (BMI) is positively associated with BMD (10), the risk of fracture is not reduced with increasing BMI (11–13). More recent findings actually associate abdominal obesity (14–16) and T2D (4, 8, 17) with increased risk of fractures.

Some studies indicate that a diet rich in protein may increase bone turnover. In 2015, Kerstetter et al. found that 18 months of whey protein isolate supplements (45 g/d) versus isocaloric maltodextrin induced an increase in CTX and preserved fat-free mass in elderly men and women with BMI from 19 to 32 kg/m^2^ (18). In 2017, Heer et al. found 60 days of increased protein intake (1.45 g/kg/d + 0.72 g/d branched chain amino acids versus 1 g protein/kg/d) to increase P1NP and CTX, N-terminal telopeptide (NTX) and Tartrate-resistant acid phosphatase (TRAP) in bedridden women (19).

Studies on long-term protein supplements and bone are primarily performed on subjects with osteoporosis - a disease in which the increased fracture risk is based on elevated bone turnover and thus very different from obesity and T2D (20). The effect of whey protein per se on bone turnover markers is only known in the acute setting, in which bone turnover is lowered which is the case for all macronutrients (21). Whey protein is known for its insulinotropic abilities compared to other protein sources in the acute settings (22). However, the long-term effect of protein intake on bone turnover in insulin resistant subjects is not elucidated. In the present study we investigated whether long-term whey protein intake stimulate bone turnover.

Using data from the MERITS trial (23), we examined if increased intake of whey protein for 12 weeks would increase bone turnover in prediabetic subjects with abdominal obesity.

Moreover, we aimed to examine the association between change in bone turnover and insulin resistance.

## Materials And Methods

### Study Design and Participants

The present study was part of the MERITS trial, described in detail previously (23). The trial was a controlled intervention study in which 65 subjects with abdominal obesity completed 12 weeks of dietary supplement with either whey protein (WP) or maltodextrin (MD) in combination with either high (HiFi) or low (LoFi) dietary fiber diet. The primary aim of the MERITS study was to assess changes within lipid metabolism, whereas secondary aims included changes in bone turnover.

We recruited 73 subjects with age ≥40 years with abdominal circumference ≥94 cm (men) or ≥80 cm (women). Exclusion criteria included osteoporosis, diabetes, severe renal, cardiovascular, or psychiatric illness or medical treatment with hormonal replacement therapy or corticosteroids. We allowed for the continued use of regular medication, including vitamin D and calcium supplements, if no changes occurred during the trial or three months prior to inclusion.

By blocks of eight, we randomized the participants by age and sex into one of four groups: WP + high fiber (WP-HiFi), WP + low fiber (WP-LoFi), maltodextrin + high fiber (MD-HiFi), or maltodextrin + low fiber (MD-LoFi).

For a period of 12 weeks, the participants received powder supplements twice daily (2 x 30 g of WP or MD). Furthermore, participants were to substitute bread and cereal products of their normal diet with test products (bread and cereals) of either high or low fiber content aiming at a fiber intake of 30 g (HiFi) or 10 g (LoFi) fiber per day from test products.

Arla Foods Ingredients Group P/S (Viby, Denmark) provided the WP hydrolysate (Lacprodan^®^ HYDRO.REBUILD) and MD (Glucidex^®^ 19). Lantmännen (Vaasan bakery, Vilnius, Lithuania)/Lantmännen Cerealia AB (Järna, Sweden) provided bread and cereal products.

The test products were iso-energetic (WP and MD, HiFi and LoFi). Data on nutritional composition of the test products are given elsewhere (23). Calcium content within powders was negligible. Participants were to refrain from any change in physical activity or any further change in dietary intake during the study, as no change in weight was intended.

The HiFi bread and cereals consisted of enzyme-treated wheat bran and refined wheat, while LoFi products were based only on refined wheat. Enzyme treatment was performed by DuPont Industrial Biosciences Aps (Brabrand, Denmark) to increase fiber solubility for improved baking properties.

The study was conducted in accordance with the Declaration of Helsinki of 1975 (as revised in 1983), approved by the Central Denmark Region Committees on Health Research Ethics (Journal no. 1-10-72-370-15) and registered at ClinicalTrials.gov (NCT02931630).

### Study Visits

At trial initiation, participants attended the clinic after an overnight fast (minimum 10 h) by means of car or public transportation. Blood was drawn from an antecubital vein between 7.00 and 8.30 AM. A Whole-body Dual-Energy X-ray Absorptiometry (DXA) scan (Hologic Horizon A scanner, Hologic, Inc., Massachusetts, USA using Apex System Software version 5.6.0.5) was performed to assess body composition. Whole body BMD excluding the head region is reported in this manuscript. Participants collected 24-h urine samples using 3L containers in cooling bags, which were analyzed for carbamide and calcium. The procedures were repeated after 12 weeks of dietary intervention.

Participants picked up the intervention products at the test site with regular intervals.

Dietary adherence was assessed by self-reported 3-day weighed dietary records at the beginning, middle and end of the trial, and by measuring urinary carbamide and plasma alkylresorcinols (markers of dietary protein and fiber intake, respectively).

### Blood Analysis

Blood samples were centrifuged at 2000 × *g* for 15 min at 4 °C, immediately frozen at −20 °C and moved to −80 °C within 8 h. All fasting values were calculated as the mean of three consecutive fasting blood samples.

Plasma glucose was measured on Cobas c111-system by standard enzymatic colorimetric assays using commercial kits (cat. 04657527, Roche Diagnostics GmbH). Intra-/inter-assay precision were between 0.8–1.1% and 0.5–0.6%. Plasma insulin was measured by ELISA technique using commercial kits (cat. K6219, Dako Denmark A/S) with intra-/inter-assay precision of 5.1–7.5% and 4.2–9.3%.

Serum CTX, P1NP, and PTH were measured on Cobas 6000 e 601 system using ELISA sandwich immunometric assays method (Roche Diagnostics GmbH). Intra-/inter-assay precision was 1.4–3.2%/1.9–3.4% for P1NP, 1.2–4.7%/1.5–5.7% for CTX, and 1.1–2.0%/1.7–3.4% for PTH.

Plasma ionized calcium, phosphate, magnesium, alkaline phosphatase, 25(OH)D, glomerular filtration rate (GFR) and urinary calcium and carbamide were analyzed at the Department of Clinical Biochemistry at Aarhus University Hospital, Denmark (DS/EN ISO 15189:2013 approved).

All analyses were pre-specified at the project origin.

### Statistical Analysis

The final analysis included only participants who completed the trial.

Baseline values are displayed as means with SD unless otherwise indicated.

Two-factor ANOVA was used to assess any effect or interaction of protein or fiber intake on markers of bone turnover. Any significant effect was followed by a pairwise comparison of groups corrected for multiple comparisons by Tukey-Kramer method. Estimates were adjusted for age and sex.

Linear regression analysis was used to determine associations between change in insulin resistance and bone turnover markers in all subjects. Assumptions of linear regression were checked using scatter plots, QQ plots and histograms of the residuals. The regression analyses were not corrected for multiple testing due to the exploratory nature of the study and the intent to generate new hypotheses.

Normality and variance across groups was checked by diagnostic plots of the residuals. If these were not met, the dependent variable was log-transformed.

Homeostasis model assessment of insulin resistance was calculated by the formula: Fasting plasma glucose (mmol/L) x fasting plasma insulin (mU/L)/22.5.

All statistics were performed using STATA/IC 15.1 (StataCorp LP College Station, TX, USA).

Power calculations for sample size were based on expected change in postprandial triglycerides, which was the primary aim of the MERITS trial (23).

## Results

Baseline characteristics by randomization group are listed in **Table 1**. Further baseline values of the various outcomes are displayed in **Table 2**.

**Table 1:** Baseline characteristics by randomization group.

	WP-LoFi	WP-HiFi	MD-LoFi	MD-HiFi
Subjects (n)	15	16	16	17
Age (years)^1^	67 (60,69)	65 (59, 69)	62 (58, 68)	64 (56, 67)
Sex (male/female)	9/6	7/9	8/8	7/10
Smoking (n)	2	0	0	2
BMI, kg/m^2^	28 (4)	29 (2)	30 (4)	29 (4)
Metabolic syndrome (n)	10	9	8	7
Obesity, BMI>30 (n)	6	5	9	5
Postmenopausal (n)	4	8	6	8
p-calcium, ionized (mmol/L)	1.27 (0.04)	1.28 (0.03)	1.26 (0.04)	1.27 (0.03)
p-magnesium (mmol/L)	0.86 (0.06)	0.86 (0.04)	0.85 (0.05)	0.88 (0.05)
p-phosphate (mmol/L)	0.94 (0.16)	1.00 (0.12)	0.91 (0.24)	0.96 (0.15)
p-alkaline phosphatase (U/L)	77.1 (20.0)	84.9 (26.7)	72.3 (18.5)	75.8 (20.9)
p-eGFR (ml/min)	84.5 (5.8)	77.9 (10.7)	82.8 (9.2)	82.1 (8.5)

Values are means (SD) unless otherwise specified. ^1^ Median (25th and 75th centile).

WP, whey protein; MD, Maltodextrin; LoFi, low fiber; HiFi, high fiber; eGR, estimated glomerular filtration rate.

**Table 2:** Baseline values and changes following 12-weeks of dietary intervention.

	WP-LoFi	WP-HiFi	MD-LoFi	MD-HiFi	Two-factor ANOVA, p value		
					Protein group	Fiber group	Inter-action
P1NP, baseline^1^(μg/L)	47.2 (32.9, 65.7)	44.8 (35.1, 57.6)	36.8 (30.3, 45.9)	56.5 (40.1, 61.3)			
P1NP, change(μg/L)	-0.43 (11.47)	0.51 (7.35)	1.43 (5.99)	-1.93 (10.47)	0.88	0.53	0.37
P1NP, change (adj.) (μg/L)^2^	-0.24 (2.38)	0.26 (2.29)	2.22 (2.36)	-2.26 (2.27)	0.93	0.37	0.26
CTX, baseline^1^(μg/L)	0.38 (0.23, 0.53)	0.37 (0.30, 0.49)	0.34 (0.25, 0,38)	0.34 (0.29, 0.47)			
CTX, change(μg/L)	-0.02 (0.10)	-0.01 (0.07)	-0.01 (0.08)	0.02 (0.07)	0.23	0.30	0.69
CTX, change (adj.)(μg/L)^2^	-0.02 (0.02)	-0.01 (0.02)	-0.01 (0.02)	0.02 (0.02)	0.23	0.32	0.71
PTH, baseline^1^ (pmol/L)	4.0 (3.5, 5.5)	4.2 (3.3, 4.7)	3.7 (3.4, 4.8)	3.9 (3.4, 4.9)			
PTH, change(pmol/L)	-0.1 (0.7)	-0.0 (0.8)	0.2 (0.8)	0.1 (0.6)	0.15	0.96	0.54
PTH, change (adj.)(pmol/L)^2^	-0.14 (0.17)	-0.07 (0.16)	0.43 (0.17)	-0.02 (0.17)	0.06	0.26	0.12
HOMA-IR, baseline	1.92 (0.89)	1.83 (0.83)	2.15 (0.81)	1.50 (0.82)			
HOMA-IR, change	0.05 (0.76)	-0.12 (0.36)	0.27 (0.53)	0.10 (0.37)	0.10	0.25	0.98
U-calcium, baseline (mmol/d)	4.12 (1.33)	3.88 (2.58)	3.63 (1.50)	4.36 (2.54)			
U-calcium, change (mmol/d)	1.12 (1.29)*	0.34 (1.09)	-0.03 (1.61)	-0.28 (1.83)	**0.03**	0.23	0.50
U-carbamide, baseline (mmol/d)	382 (103)	302 (117)	363 (150)	320 (132)			
U-carbamide, change (mmol/d)	208 (33)*^a^	212 (30)*^a^	-36 (31)^b^	60 (29)*^b^	**<0.01**	0.11	0.13
T-score, baseline	-0.9 (1.5)	-0.96 (1.0)	-0.6 (1.05)	-1.15 (1.17)			
T-score, change	0.06 (0.14)	-0.03 (0.12)	0.10 (0.28)*	0.05 (0.15)	0.25	0.20	0.74
BMD, baseline	1.09 (0.14)	1.07 (0.10)	1.10 (0.11)	1.05 (0.12)			
BMD, change	0.01 (0.01)	-0.003 (0.01)	0.005 (0.02)	0.005 (0.01)	0.31	0.33	0.23
25(OH)D, basline (nmol/L)	64.1 (26.4)	74.8 (26.7)	60.8 (25.3)	75.4 (22.5)			
25(OH)D, change (nmol/L)	2.3 (12.6)	1.1 (13.9)	11.8 (24.9)*	-3.8 (16.9)	0.71	0.06	0.12

All values are means (SD) unless otherwise stated. Values within a row with different superscript letters are statistically different (p<0.05). P-values <0.05 are displayed in bold.

^1^Median (25th and 75th centile).

^2^Adjusted for change in 25(OH)D. Values given as mean change (SE),

WP, whey protein; MD, Maltodextrin; LoFi, low fiber; HiFi, high fiber; P1NP, procollagen type 1 N-terminal propeptide; CTX, C-terminal cross-linked telopeptide type 1 collagen; PTH, parathyroid hormone; HOMA-IR, Homeostatic Model Assessment of Insulin Resistance; BMD, bone mineral density; 25(OH)D, 25-hydroxy vitamin D.

Study flow chart and dietary intake reports have previously been reported (23). In brief, 64 participants were included in the present study (one participant excluded due to osteoporosis). Two subjects dropped out due to dislike of test products. Test products were otherwise well tolerated. By self-reported, weighed, 3-d dietary intake reports, compliance was deemed high. The mean protein intake in the WP groups was 141.6 (16.5) g/d versus 86.8 (18.1) g/d in MD groups. Mean fiber intake was 34.6 (4.9) g/d in HiFi groups versus 16.0 (5.2) g/d in LoFi groups. The reports were supported by levels of plasma alkylresorcinol and urinary carbamide as markers of fiber and protein intake (23).

**Table 2** shows baseline values and changes following the 12-week intervention within the four groups. Twelve weeks of high protein and/or high fiber intake did not affect levels of P1NP or CTX. Likewise, we did not find any change in PTH. However, as 25(OH)D is known to strongly affect PTH levels, we adjusted for change in 25(OH)D, which modified the results, showing a negative trend between protein intake and PTH levels (p=0.06).

As expected, we found an increased u-calcium and u-carbamide in both protein groups.

There was a non-significant negative association (p=0.06) between change in dietary fiber intake and change in 25(OH)D.

The intervention did not affect insulin resistance within the four groups (additional data on insulin resistance reported elsewhere (24)). **Table 3** displays the association between change in insulin resistance and change in bone turnover markers by linear regression analysis in all subjects. There was a positive association between change in HOMA-IR and PTH, while changes in P1NP and CTX did not associate to changes in HOMA-IR. The change in PTH was not associated with changes in CTX or P1NP (p=0.24, p=0.26).

**Table 3:** Multiple linear regression analysis of change in insulin resistance by HOMA-IR and change in bone markers.

	Coefficient	95% CI	p-value
ΔP1NP	-0.01	(-0.02, 0.01)	0.29
ΔP1NP adjusted (sex)	-0.01	(-0.02, 0.01)	0.33
ΔP1NP adjusted (sex, 25(OH)D)	-0.01	(-0.02, 0.01)	0.27
ΔCTX	0.42	(-1.27, 2.11)	0.62
ΔCTX adjusted (sex)	0.29	(-1.43, 2.01)	0.74
ΔCTX adjusted (sex, 25(OH)D)	0.25	(-1.48, 1.99)	0.77
ΔPTH	0.19	(0.01, 0.37)	**0.038**
ΔPTH adjusted (sex)	0.19	(0.02, 0.37)	**0.033**
ΔPTH adjusted (sex, 25(OH)D)	0.32	(0.01, 0.63)	**0.042**

P1NP, procollagen type 1 N-terminal propeptide; CTX, C-terminal cross-linked telopeptide type 1 collagen; PTH, parathyroid hormone; 25(OH)D, 25-hydroxy vitamin D. P-values <0.05 are displayed in bold.

## Discussion

It is a common belief, that bone health is influenced by dietary habits. In the standard treatment of osteoporosis (25), a bone healthy diet aiming at avoiding calcium and vitamin D deficiency, is recommended. However, when minerals and vitamins are accounted for, the effect of everyday meals on bone health remains sparse. The introduction of circulating bone turnover markers has provided a simple way of assessing the acute bone response to various stimuli. Ingestion of food suppresses the immediate bone turnover (21, 26–28), however the long-term effect of daily meals of varying composition on bone health is not well characterized.

Diets of increased protein content are generally advised to preserve bone mass (29–31), although this recommendation is primarily based on pooled evidence from studies showing no adverse effects and at best, a modest reduction in fracture risk (32–34). Most studies are performed in healthy subjects or subjects with postmenopausal osteoporosis.

In type 2 diabetes, abdominal obesity and metabolic syndrome, bone turnover is low - in contrast to postmenopausal osteoporosis, where bone turnover is increased. Fracture risk is however increased, and the low bone turnover is suggested to accumulate microfractures and make the bone more fragile. Thus, increasing bone turnover in type 2 diabetes, abdominal obesity and metabolic syndrome may be beneficial for bone health. Very little research, if any, explore the long-term effects of diet and especially enhanced protein intake on bone within weight stable subjects with type 2 diabetes, abdominal obesity or metabolic syndrome.

We hypothesized that the increase in protein intake would lead to increased bone turnover. This was however not the case. Both bone formation and degradation markers remained unchanged after 12 weeks of whey protein versus placebo. Although, we did observe a negative trend for an association between protein intake and PTH levels (p=0.06). In normal weight subjects, low protein diets are known to lead to secondary increase in PTH which is believed to relate to reduced intestinal calcium absorption and increased bone turnover (35). Likewise, in healthy women increased protein intake is reported to lead to decreased PTH (36), which is consistent with our findings.

We report an increase in urinary calcium excretion in the protein group. It is well known that increased protein intake induces increased urinary calcium excretion (37). Increased calcium absorption from the gut (seemingly independent of calcium intake) and bone degradation have been proposed as potential mechanisms, although this remains widely debated. An increased absorption of calcium from the gut may explain our borderline significant negative association between protein intake and PTH. In the current study, the calcium intake from test products was negligible, as WP hydrolysate only added 6 mg/d of extra calcium (data not displayed). The finding of an increased urinary calcium excretion in the protein group concurs with existing evidence.

Thus, a protein-rich diet in subjects with abdominal obesity appears to affect calcium homeostasis similarly to normal weight individuals.

The secondary hypothesis of the study was that insulin resistance and bone turnover are inversely associated. We did however not observe any difference in insulin sensitivity by HOMA-IR or bone markers between groups. In a similar dietary intervention study on type 2 diabetic subjects a high protein diet improved HbA1c but not HOMA-IR, indicating that primarily postprandial insulin sensitivity was affected (38), which is not reflected in HOMA-IR. In our study, postprandial insulin sensitivity assessed by Matsuda index was however unaffected (data shown elsewhere (24),). As the present study was primarily designed to identify changes in the lipid metabolism and not in bone markers or insulin resistance, we may not have had enough subjects to obtain significant changes, which naturally is a weakness of this *post hoc* study.

As formerly mentioned, insulin resistant subjects with type 2 diabetes are reported to have low bone turnover (3). We previously reported associations between decreased levels of bone turnover markers and insulin resistance in non-diabetic subjects with abdominal obesity (5). In the current study, we hoped to see bone turnover increase in subjects that became more insulin sensitive, and potentially *vice versa*, assuming that the regulatory mechanism is reversible. This was not the case, as we did not see any association between change in P1NP or CTX with change in HOMA-IR. We did find an association between increase in PTH and increase in insulin resistance in all subjects independent of the dietary intervention. This finding is interesting as it aligns with previous reports were hyperparathyroidism is associated with diabetes and insulin resistance (39). How the association between PTH and insulin resistance relates to a reduced bone turnover in patients with type 2 diabetes is not well understood, but a possible mechanism may be elicited by osteocytic dysfunction with excess production of sclerostin (8).

We report the findings of a *post hoc* analysis of a randomized controlled trial. Thus, no power calculation was performed on bone turnover markers which is a limitation of the current study. As we did not observe a trend on changes in markers of bone turnover, we do not expect that a larger sample would have revealed any differences in CTX or P1NP between the groups. It is possible that a population of increased insulin resistance and lower bone turnover at baseline, such as T2D subjects, would have increased the chances of detecting changes in bone turnover. We assume our study duration is too short to induce changes in BMD. Furthermore, we have not measured BMD at regional sites (e.g. lumbar spine and hip). We found a nonsignificant increase in 25(OH)D within the LoFi groups (p=0.06), that was most likely due to lower baseline values in these groups. Any seasonable variation during the 13 months the trial ran (from May 2016 to June 2017), was expected similar within groups because of the continuous block randomization.

## Conclusion

The current study did not find an effect of long-term supplementation of dietary protein or fiber on bone turnover in subjects with abdominal obesity. PTH tended to associate negatively with protein intake, although bone turnover markers remained unaffected. A high protein intake induced increased urinary calcium excretion, unrelated to increased calcium intake. We did find an association between measures of insulin resistance and levels of circulating PTH levels, which supports the hypothesis that insulin resistance may be key to understand the low bone turnover and increased bone fragility observed in subjects with T2D.

More long-term studies on diet and the bone turnover of subjects with type 2 diabetes, abdominal obesity and metabolic syndrome are needed.

## Data Availability Statement

The raw data supporting the conclusions of this article will be made available by the authors, without undue reservation.

## Ethics Statement

The studies involving human participants were reviewed and approved by Central Denmark Region Committees on Health Research Ethics (Journal no. 1-10-72-370-15). The participants provided their written informed consent to participate in this study.

## Author Contributions

RF-N, ER, SG, and KH conceived and designed the study. RF-N and ER conducted the study. RF-N, PV, and JS-L analyzed the data. RF-N wrote the initial manuscript. All authors critically reviewed and approved the final manuscript.

## Funding

The study was supported by a grant from Innovation Fund Denmark - MERITS (4105-00002B) and Steno Collaborative Grant, Novo Nordisk Foundation, Denmark (Grant no. NNF18OC0052064).

## Conflict of Interest

The authors declare that the research was conducted in the absence of any commercial or financial relationships that could be construed as a potential conflict of interest.

## Publisher’s Note

All claims expressed in this article are solely those of the authors and do not necessarily represent those of their affiliated organizations, or those of the publisher, the editors and the reviewers. Any product that may be evaluated in this article, or claim that may be made by its manufacturer, is not guaranteed or endorsed by the publisher.
